# Role of Type I Interferons on Filovirus Pathogenesis

**DOI:** 10.3390/vaccines7010022

**Published:** 2019-02-20

**Authors:** Beatriz Escudero-Pérez, César Muñoz-Fontela

**Affiliations:** 1 Bernhard Nocht Institute for Tropical Medicine, 20359 Hamburg, Germany; munoz-fontela@bnitm.de; 2 German Center for Infection Research, Partner Site, 20359 Hamburg, Germany

**Keywords:** Interferon, filovirus, immune responses, pathogenicity

## Abstract

Filoviruses, such as Ebola and Marburg virus, encode viral proteins with the ability to counteract the type I interferon (IFN-I) response. These IFN-I antagonist proteins are crucial to ensure virus replication, prevent an antiviral state in infected and bystander cells, and impair the ability of antigen-presenting cells to initiate adaptive immune responses. However, in recent years, a number of studies have underscored the conflicting data between in vitro studies and in vivo data obtained in animal models and clinical studies during outbreaks. This review aims to summarize these data and to discuss the relative contributions of IFN-α and IFN-β to filovirus pathogenesis in animal models and humans. Finally, we evaluate the putative utilization of IFN-I in post-exposure therapy and its implications as a biomarker of vaccine efficacy.

## 1. Filoviruses Counteract the Type I Interferon Response

Marburg virus (MARV, species *Marburg marburgvirus*) and Ebola virus (EBOV, species *Zaire ebolavirus*) are non-segmented, negative-strand RNA viruses that belong to the *Filoviridae* family. They are prominent human pathogens that can cause severe hemorrhagic fever, with case-fatality rates of up to 90% in humans. The pathophysiology of the Ebola and Marburg virus diseases is characterized by systemic virus dissemination and dysregulation of the host immune response, which is partially responsible for the multiorgan failure that characterizes the late stages of a fatal disease [1,2].

The biological mechanisms behind the high pathogenicity of these viruses in humans are poorly understood, but likely rely on two factors: (i) the capacity of the host to control viral replication, and (ii) the capacity of the virus to counteract the host defense mechanisms. Indeed, a poor outcome from Ebola virus disease (EVD) is correlated with high levels of viremia [3,4], suggesting that the ability of the virus to subvert host immune responses, replicate in various cell types, and reach the bloodstream plays an important role in fatal filovirus infection.

The innate immune system is equipped with microbial sensors—namely, pattern-recognition receptors (PRRs) that respond to different pathogen-associated molecular patterns (PAMPs), one of which is viral RNA [5,6,7]. Activation of PRRs leads to the production of interferons (IFN), the main antiviral cytokines. In turn, the binding of IFN to its receptors induces the transcription of multiple interferon-stimulated genes (ISG), whose protein products have antiviral activity and immunomodulatory effects. 

IFNs are typically divided among three classes: Type I IFN (IFNα/β), Type II IFN (IFNγ), and Type III IFN (IFNλ). In general, type I and II IFN are responsible for regulating and activating the immune response. Expression of type I IFN (hereafter referred to as IFN-I) can be induced in almost any cell type upon recognition of PAMPs, whereas type II IFN (IFN-II) is induced by cytokines like IL-12, and its expression is restricted to immune cells, such as T cells and natural killer (NK) cells [8]. Although IFN-I and IFN-II use distinct transmembrane receptors to initiate their signaling cascades, they converge upon the Janus kinase (JAK)–signal transducer and activator of transcription (STAT) pathway. When IFNs bind to specific cell-surface receptors, they activate a cascade of signal transduction and transcription (STAT) proteins. This leads to the transcription and synthesis of oligoadenylate synthetase (OAS); double-stranded, RNA-associated protein kinase (PKR); IFN regulatory factor (IRF) 1; and other proteins, creating an antiviral state in infected and bystander cells [9,10]. A number of viruses, filoviruses among them, have acquired means of subverting or evading the IFN-I response as part of their replication strategy [11,12]. 

EBOV has seven genes coding for eight major viral products, two of which (VP24 and VP35) have been shown to act as IFN-antagonist proteins. Interestingly, the corresponding proteins with IFN antagonist function, in the case of MARV, are VP35 and VP40. Below, we provide a summary of the molecular mechanisms by which VP35, VP24, and VP40 subvert the IFN-I immune function. For a detailed discussion of these molecular mechanisms, the reader is here directed to recent excellent reviews [2,12]. 

### 1.1. VP35

Mammalian cells infected with RNA viruses recognize the intruder through retinoic acid-inducible gene I (RIG-I)-like receptors (RLR) or via endosomal toll-like receptors (TLRs). In the case of filoviruses, a blockade of the RIG-I pathway results in enhanced susceptibility to EBOV, suggesting that EBOV recognition and innate immune responses require RIG-I [13]. Therefore, it is not surprising that both EBOV and MARV encode an IFN antagonist protein—namely, VP35—that primarily targets RIG-I. VP35 proteins are double-stranded RNA (dsRNA)-binding proteins that are essentially co-factors of the filovirus polymerase complex [14,15]. In addition to its role on virus replication, VP35 displays RNA silencing activity, targets RIG-I signaling, and inhibits PKR function [11,16,17]. Through these mechanisms, VP35 is able to inhibit both IFN-I signaling and production.

Experiments in cell culture have already indicated that suppression of RIG-I activity is critical for filovirus replication. For example, pre-activation of RIG-I before EBOV infection resulted in a significant reduction in EBOV replication [18]. Further research has demonstrated that both EBOV and MARV VP35 proteins are able to counteract the antiviral function of RIG-I via different mechanisms. VP35 inhibits the RIG-I pathway at several levels, through interaction with cellular kinases IKKε and TBK-1, and through interaction with the SUMOylation machinery [19,20]. Moreover, VP35 can inhibit the function of RIG-I through the adaptor protein PACT (protein activator of the interferon-induced protein kinase, PKR), which was first described to interact with and activate PKR [21]. PACT induces the ATPase activity of RIG-I; thus, through the sequestering of PACT, VP35 can reduce RIG-I activation [22]. To exert these functions, VP35 relies on its IFN inhibitory domain, as shown by the fact that mutations in this domain abrogate the ability of the viral protein to target RIG-I, which results in virus attenuation in cell culture and in a mouse model [23,24,25,26,27]. The importance of RIG-I targeting for EBOV pathogenesis was illustrated by experimental infection of mice lacking expression of mitochondrial antiviral signaling protein (MAVS), a RIG-I adaptor protein.. In these mice, the absence of RIG-I signaling resulted in significantly higher susceptibility to EBOV infection than their wt counterparts [13]. 

Importantly, VP35 also impairs IFN-I signaling by antagonizing the activation of PKR. In uninfected cells, PKR is present at low levels, and its expression is substantially increased with IFN-I production. During viral infection, PKR binds dsRNA and phosphorylates the translation initiation factor eIF-2 (eIF-2α), which then abrogates protein synthesis to inhibit viral replication. When EBOV VP35 binds to PKR, it prevents its antiviral effect [28]. The ability of VP35 to target PKR seems to be cell- and species-specific, as MARV VP35 can inhibit PKR activation in 293T cells but not in human glioblastoma U-251-MG cells, or in a cell line derived from Rousettus bats (*Rousettus aegyptiacus*) [28], a reservoir of MARV [29]. 

Interestingly, the potency of VP35 as IFN-I inhibitor does not correlate with filovirus pathogenicity. For example, despite the fact that EBOV and MARV display similar virulence, EBOV VP35 has been shown to be a more potent IFN-I inhibitor than MARV VP35 in a human monocytic cell line, a feature that has been partially attributed to the ability of EBOV VP35 (but not MARV VP35) to cap the ends of viral dsRNA [30]. Perhaps more illustratively, EBOV but not *Reston ebolavirus* (RESTV) is highly pathogenic for humans, despite the fact that both EBOV and RESTV VP35 share a similar ability to block IFN-I in vitro [31]. A possible confounding factor is the species origin of the cells utilized in these in vitro studies. For example, several differences in the IFN response between human and bat cells have been shown to occur during filovirus infections. More specifically, MARV has been shown to induce a potent innate immune response in *Rousettus* bat cells, which was generally stronger than that in human cells. Also, while bat IFN-I can induce an antiviral state in both bat and human cells, IFN-γ of bat origin controls filovirus infection in bat cells, but not in human cell lines [32].

### 1.2. VP24 and VP40

The MARV VP40 and EBOV VP24 proteins share the ability to inhibit the IFN-I signaling pathway, albeit through different mechanisms. MARV VP40 blocks Jak1 function and inhibits the tyrosine phosphorylation events downstream of the IFN-α/β receptor [33,34]. In the case of EBOV VP24, the effect is dependent on the inhibition of karyopherin-mediated nuclear translocation of STAT1 [34]. Together with STAT2 and IRF9, STAT 1 is a component of the IFN-stimulated gene factor 3 (ISGF3), which activates the transcriptional activation of hundreds of effector genes harboring IFN-stimulated response elements (ISREs) in their promoters [35]. The action of both proteins therefore leads to reduced transactivation of IFN-I stimulated genes (ISGs), precluding the establishment of the IFN-I antiviral state in infected and neighboring cells [34,36]. Similar to VP35, loss of VP24 function is crucial for EBOV virulence in rodents [26]. As opposed to humans and non-human primates (NHP), rodents are entirely resistant to filovirus infection, but this resistance can be overcome by abrogation of the IFN-I response [37]. However, several passages of EBOV in suckling mice results in the selected emergence of EBOV rodent-adapted variants, which harbor coding changes in the nucleoprotein (NP), VP35, glycoprotein (GP), VP24, and polymerase (L) that are highly pathogenic in mice [26]. Adaptation in mice requires an insertion as well as an amino acid change in VP24, suggesting that this protein is crucial in the increase of EBOV virulence in the rodent model.

### 1.3. Blocking the IFN-I Response in Target Cells

Since antigen-presenting cells (APC)—in particular, dendritic cells (DCs) and macrophages—are primary filovirus target cells [37,38], some studies have been conducted to evaluate the effect of filovirus IFN-antagonist proteins on APC function. Using DCs derived from blood monocytes, two studies have demonstrated that EBOV replicates in human DCs without inducing signs of maturation—namely, expression of T-cell co-stimulatory molecules and cytokine production [39]. Although these studies did not directly address the relationship between VP24 and VP35 function and DC maturation, others have shown that expression of VP24 and VP35 alone in DCs inhibits IFN-I signaling, and results in impaired maturation and dysregulation of T-cell-mediated immunity [17,40,41]. Monocyte-derived inflammatory DCs, however, represent only a subset of the wide variety of DCs in vivo (see [42] for an excellent review); thus, it will be important to determine the DC tropism of filoviruses in vivo, in particular at early time points after infection. In this regard, a recent study in a mouse model indicates that while EBOV can readily infect inflammatory DCs, it does so independently from whether or not those cells harbor functional IFN-I receptors [37]. This scenario differs from that of influenza infection, where the permissiveness of DCs to infection was linked to their inability to respond to IFN-I [43]. Of note, in the mentioned mouse study, langerin-expressing DCs were spared from EBOV infection, which is consistent with the overall resistance of these cells to fusion with enveloped viruses [44,45]. Understanding filovirus antagonism in infected DCs may be also highly relevant to determine how filoviruses modulate the initiation of host-adaptive immune responses. DCs are the only cell type capable of priming naïve cognate T-cells, and their capacity to do so depends to some extent of IFN-I-induced maturation [46]. Moreover, IFN-I bridges innate and adaptive immune responses through the modulation of natural killer (NK) cell activation [47], cross-presentation [48], and antibody production [49] among others.

The ability of filoviruses to block IFN-I, together with other observations like lymphocyte apoptosis during infection, suggests an association between immune inhibition and filovirus pathophysiology. However, human disease data gathered during the West African 2013–2016 EVD epidemic shows strong and sustained T-cell activation [50], proving a challenge to this model. 

## 2. The Role of IFN-I over the Course of Filovirus Disease

### 2.1. Filovirus Infection in Mouse Models

Throughout the years, several animal models have been developed, with the main goal of testing filovirus vaccines and therapeutics, but also to study filovirus pathogenesis and immunity. Rodent models, in particular laboratory mice, are most valuable tools for dissecting complex biological mechanisms, due to the availability of transgenic and gene-knockout strains. However, mice are entirely resistant to filovirus disease. To circumvent this problem, mouse-adapted filovirus strains have been developed; however, the number of adapted strains and variants is limited, therefore precluding comparative studies. Alternatively, the mouse model has been modified to allow non-adapted filovirus replication. In this regard, mice deprived of their endogenous IFN-αβ response are susceptible to lethal EBOV, *Sudan ebolavirus* (SUDV), and MARV infection. Furthermore, wild-type BALBc mice treated with anti-IFN-αβ antibodies have been shown to be susceptible to lethal infection with EBOV, mouse-adapted EBOV, and SUDV [51]. These findings are interesting, since they strongly indicate that EBOV is able to overcome initial barriers to infection, but probably cannot effectively replicate in cells in which IFN-α has already induced an antiviral state. These results are in agreement with our unpublished observations that, despite failing to cause disease, EBOV can replicate for several days in the respiratory mucosa of C57BL/6 mice (unpublished data). In fact, we have observed that EBOV can replicate in many IFN-competent mouse cells, including DCs, macrophages, and hepatocytes [37]. In a mouse chimera study, where IFN deficiency was restricted to the stromal compartment, the mice mounted, hematopoietic-driven, adaptive immune responses, but 50% of subjects succumbed to infection, showing evidence of inflammation, liver failure, and shock [37]. In this model, depletion of CD8 and CD4 T-cells resulted in systemic virus dissemination and 100% lethality. Increasing the genetic diversity in mice also resulted in loss of protection [52], indicating that many other factors, in addition to the potency of the murine IFN-I response, are involved in the filovirus resistance of rodents. In fact, different humanized mouse models with limited adaptive immune responses (i.e., T-cell oligoclonality, immunoglobulin isotype switch defects) but intact IFN-I are highly susceptible to non-adapted filoviruses [53,54,55]. These findings suggest that IFN-mediated effects on the host adaptive immune response may play an important role on filovirus immunity in mice. It would be highly interesting to explore the relative contribution of IFN-I effects on adaptive versus innate immunity to filovirus resistance in rodent models.

### 2.2. Filovirus Infection in Non-Human Primates

Non-human primates (NHP) are the gold-standard animal models for studying filovirus pathogenesis. Specifically, rhesus and cynomolgus macaques are highly sensitive to filovirus infection, and faithfully recapitulate many of the disease manifestations observed in humans, including multiorgan failure, shock, and hemorrhage [38]. However, rather than showing defects on the IFN-I response, NHP infected with EBOV or MARV showed that IFN-I-stimulated genes were strongly upregulated in circulating immune cells at early time points after infection [56]. Indeed, in EBOV- and MARV-infected macaques, IFN-α is present in a 60–100-fold higher concentration compared to other acute viral infections [56,57]. Treatment of EBOV-infected NHP with IFN-α on the day-of challenge did not demonstrate a clinical benefit [58]. However, IFN-α treatment has shown efficacy in rescuing EBOV-infected NHP, in combination with monoclonal antibody therapy [59]. 

Conversely, filovirus-infected NHP produce little IFN-β [56,57], suggesting perhaps that in NHP, the antagonistic action of VP35 is stronger compared to that of VP24. In agreement with this hypothesis, post-exposure siRNA-based targeting of VP35 rescued 100% of macaques infected with EBOV and SUDV [60,61]. In this regard, it would be interesting to discern whether targeting of VP35 in vivo influences the dynamics of IFN-β production, or whether the effect of siRNA therapy is solely dependent on targeting the polymerase co-factor activity of VP35. Post-exposure treatment with IFN-β resulted in significant delayed time of death in macaques infected with either EBOV or MARV, although it failed to alter mortality [62]. These results encouraged the therapeutic use of IFN-β, in particular as a part of combination therapy. Indeed, combined monoclonal antibody therapy with an adenovirus-vectored interferon in EBOV-infected guinea pigs provides a 100% rate of survival when administered three days after exposure [63].

### 2.3. Filovirus Infection in Humans

Similar to NHP, there it seems to be a dichotomy between the role of IFN-α and IFN-β immunity during human EVD. Clinical data collected during human outbreaks has indicated that elevated levels of circulating IFN-α, as well as upregulation of IFN-I inducible genes, is correlated with fatal EVD [64,65,66,67]. Conversely, IFN-β production has been positively correlated with survival for a small cohort of EVD patients treated in the United States [68], but not those in Africa [67]. Caution should be applied, however when evaluating clinical data collected during outbreaks. For example, patients tend to arrive at the treatment centers (ETC) at very late stages of disease, and with overt viremia. It is very likely that the physiologically relevant effect of the IFN-I response and its interaction with viral antagonists takes place during the incubation period—that is, before the patient arrives at the ETC. In addition, if, as described for influenza virus, the action of the viral antagonist proteins provides a “stealth” phase, whereby the virus is allowed to replicate efficiently [69], it is bound to happen at the natural portals of virus entry, not in peripheral blood. Thus, the elevated levels of IFN-I seen in fatal cases may be a direct reflection of uncontrolled viral loads stimulating an exaggerated IFN response. Despite this caveat, due to the magnitude and urgency of the West African outbreak, a clinical trial to test the therapeutic effect of IFN-β was conducted in Guinea. Although the results of this trial suggest that treatment with IFN β-1a may be associated with improved survival, the low number of subjects enrolled in the study and some possible confounding factors, such as differences in viral loads between treated and non-treated patients, preclude definite conclusions [70]. Despite these limitations, the data obtained provides a rationale for the consideration of IFN β-1a for further clinical evaluation, perhaps in combination with antiviral drugs [71].

Further research is clearly needed to evaluate the relative contribution of IFN-α and IFN-β to filovirus immunity, even though these studies would probably be restricted to the mouse model. In other viral infections, early IFN-β production has been correlated with protection [72,73,74], an effect that has been attributed to the hierarchical mode by which IFN-I is produced. However, the requirement of IFN-β for antiviral responses does not seem to be universal, as the pathogenicity of other viruses, such as vesicular stomatitis virus (VSV) or La Crosse virus, is not affected by the presence of IFN-β, at least in the mouse model [75,76]. Moreover, in other viral infections, such as that caused by West Nile virus, IFN-α rather than IFN-β seems to play the chief role in host protection [77]. 

## 3. Role of IFN-I in Vaccine Design

Over the last two decades, a significant amount of effort has been devoted to designing and testing experimental vaccines against filoviruses, in particular against EBOV (see [78] for a recent review). Strategies have included not only vectored vaccines, such as replication competent VSV-based vaccines and adenovirus-based vaccines, but also inactivated, DNA-based, and virus-like particle-based vaccines. Despite this variety, there is surprisingly scarce data regarding the induction of innate immune responses by these vaccines. Recombinant VSV-ZEBOV-GP, which demonstrated efficacy in a ring vaccination clinical trial performed in Guinea [79], induced strong early activation of IFN-I inducible genes in vaccines, which in turn was correlated with the strength of antibody responses. Parallel studies performed in the NHP model also demonstrated an association between toll-like receptors (TLRs), IFN-I signaling activation, and the strength of VSV-ZEBOV-GP-induced antibody responses [80]. The association between IFN-I and vaccine protection is less clear, however, in the mouse model. While IFN-stimulating adjuvants were shown to increase the immunogenicity of DNA-based EBOV vaccines [81], IFNAR^-/-^ mice were fully protected from EBOV challenge after vaccination with a non-replicating, VSV-based EBOV vaccine [82]. Especially interesting is the relationship between IFN-I and the duration and magnitude of GP-specific antibody responses, which had been previously demonstrated for influenza vaccines [49]. Of note, vaccines that signal through TLR4, an EBOV-GP ligand [83], show increased generation of neutralizing antibodies [84]. There is no current data on the relationship between vaccine-induced IFN-I production and T-cell responses. Several previous studies conducted with vaccines against other pathogens have demonstrated that the strength of vaccine-induced IFN-I correlates with antigen capture by DCs, cross-presentation of antigens, and enhanced NK function [85,86,87]. This was indeed the rationale for the use of polyinosinic–polycytidylic acid (poly IC), a potent IFN-I inducer, as adjuvant of inert and replicating vaccines [87]. It will be important, therefore, to conduct future research to evaluate the role of IFN-I in the establishment of adaptive immune responses to EBOV vaccines, which may help to better compare the correlates of protection elicited by the different available platforms. 

## 4. Conclusions 

Similar to many other RNA viruses, filoviruses encode proteins with the ability to target the IFN-I response. This suggests an arms race between filoviruses and mammalian hosts, and the necessity to allocate multiple functions to a small number of available virus proteins. However, there is an important disconnection between in vitro and in vivo studies on filovirus virulence. On the one hand, in vitro data argues for an immune-suppressive nature of infection, whereby the virus is allowed to replicate without minimum immune activation. On the other hand, filoviruses cause extraordinary levels of inflammation and immune activation in vivo. We speculate that these differences are mainly related to the timing in which in vivo experiments have been conducted. In particular, since most experiments in the best available model, the NHP, have been directed to the necessary development of vaccines and therapeutics, there is no data on the effects of IFN antagonists in the skin and mucosae at early time points after infection. These studies would probably be important to understand the mechanisms by which filoviruses establish systemic infection. For example, it is conceivable that the action of IFN antagonists early after exposure in the mucosae and skin may result in sufficient levels of virus replication to allow the virus to establish systemic disease. It will be important to determine if this is the case in NHP or in other adequate models, as well as to identify the primary cells where this is bound to occur. In this regard, it will be also relevant to conduct more experiments using natural routes of infection. Finally, it will be important to determine the role of IFN-I during vaccination, and its relationship with adaptive immune responses across the different available vaccine platforms. 
